# Prefrontal cortex interactions with the amygdala in primates

**DOI:** 10.1038/s41386-021-01128-w

**Published:** 2021-08-26

**Authors:** Elisabeth A. Murray, Lesley K. Fellows

**Affiliations:** 1 Laboratory of Neuropsychology, NIMH, Bethesda, MD USA; 2 Department of Neurology and Neurosurgery, Montreal Neurological Institute, McGill University, Montreal, QC Canada

**Keywords:** Neuroscience, Anatomy

## Abstract

This review addresses functional interactions between the primate prefrontal cortex (PFC) and the amygdala, with emphasis on their contributions to behavior and cognition. The interplay between these two telencephalic structures contributes to adaptive behavior and to the evolutionary success of all primate species. In our species, dysfunction in this circuitry creates vulnerabilities to psychopathologies. Here, we describe amygdala–PFC contributions to behaviors that have direct relevance to Darwinian fitness: learned approach and avoidance, foraging, predator defense, and social signaling, which have in common the need for flexibility and sensitivity to specific and rapidly changing contexts. Examples include the prediction of positive outcomes, such as food availability, food desirability, and various social rewards, or of negative outcomes, such as threats of harm from predators or conspecifics. To promote fitness optimally, these stimulus–outcome associations need to be rapidly updated when an associative contingency changes or when the value of a predicted outcome changes. We review evidence from nonhuman primates implicating the PFC, the amygdala, and their functional interactions in these processes, with links to experimental work and clinical findings in humans where possible.

## Introduction

The amygdala comprises a group of telencephalic nuclei that are generally believed to promote Darwinian fitness [1], and thereby increase the likelihood of an organism transmitting its genes to descendants. These nuclei are thought to bias behavioral outputs in order to exploit or explore for resources (e.g., nutrients or fluids), avoid predators, produce progeny, and learn about sensory cues that signal resources, threats, or safety [2]. Much of the information processing in these nuclei is influenced by innate (i.e., genetically stored) mechanisms responsive to either external stimuli or internal states; some of these innate mechanisms are likely primate specializations. For example, humans and many other primate species have a tendency to prefer foods high in sugar and fat [3], to attend to eyes in the faces of conspecifics [4–6], and to express defensive responses to snakes even in the absence of experience with snakes [7–9]—all products of natural selection [8, 10, 11].

The prefrontal cortex (PFC) is comprised of several subregions, including both agranular regions common to all mammals and granular regions specific to primates (see Preuss and Wise, this issue). In contrast to the innate responses linked to the amygdala, which while usually beneficial tend to be rigid, PFC function is thought to enhance behavioral flexibility. PFC subregions each make specialized contributions to cognition and behavior, based on their distinct patterns of inputs and outputs. One way to characterize PFC function generally is to say that it encodes, represents, and stores knowledge about behaviors, including the consequences of particular goals chosen and actions made in a given context [12, 13]. The representations stored in the primate-specific parts of the PFC are characterized by rapid, context-sensitive learning [14, 15], which reduces errors more effectively than the learning mechanisms common to all mammals [13].

Among the most important connections of the amygdala, axonal projections to and from the PFC contribute to both innate behavior and behavioral flexibility. Behavioral flexibility benefits from incorporating the survival-relevance of sensory inputs and behavioral outputs; and innate behavior benefits from information about the learned contexts in which benefits or threats are realized. For example, a rigid, rapid fight-or-flight response is crucial when danger looms, but may lead to opportunity costs or overt harm when deployed in an inappropriate context [16]. Thus, amygdala–PFC interactions have the potential to improve information processing in both regions. By marrying the strengths of survival instincts to the flexibility gained by nuanced processing of sensory inputs in a wide range of contexts, amygdala–PFC interactions support adaptive behaviors in dynamic and challenging situations. However, as Pine et al. [17]. have emphasized, evolutionary adaptations that provided advantages in ancestral species can cause vulnerabilities to mental illness in the present.

Aberrant amygdala–PFC connectivity has long been considered a key feature of anxiety disorders [18, 19]. This is conventionally conceived of as an imbalance, with bottom-up signaling of threat by the amygdala insufficiently dampened by top-down PFC control. This is an appealing account, in that anxiety disorders are characterized by maladaptive engagement of responses such as fear, enhanced vigilance, sympathetic arousal, and stereotyped avoidance behaviors that are poorly tuned to the current circumstances. While there is substantial neuroimaging evidence implicating the PFC, the amygdala, and their interconnections in anxiety disorders (as well as in variation in nonpathological trait anxiety) [20], a simple account of amygdala–PFC imbalance is insufficient to explain many of the research findings involving this circuitry.

In this review, we consider the role of amygdala–PFC interactions in four broad classes of behavior, all of which are known to depend on the integrity of the amygdala: approach-avoidance learning, foraging, predator defense, and social signaling. In social animals, including nonhuman primates and humans, these behaviors constitute a sophisticated repertoire for pursuing motivationally relevant goals (e.g., food or mates) in the face of potential threats, such as predation or aggression from conspecifics (see, for example, [21]). These behaviors must be deployed in a precise context and with appropriate timing to be adaptive, and amygdala–PFC interactions are critical to the optimization of this repertoire of survival-relevant behaviors. Conversely, dysfunction in these amygdala–PFC interactions may lead to maladaptive engagement of these same behaviors, with the potential to disrupt key aspects of decision-making, learning, and social behavior.

An extensive body of work deals with the neural substrates of approach–avoidance behaviors and foraging in rodents, including the role of the amygdala–PFC interactions in these species (see Rudebeck and Izquierdo, this issue). We deal only superficially with rodent research here, touching on relevant findings from rodents in the sections *Amygdala–PFC interactions in foraging* and *Amygdala–PFC interactions in predator avoidance*. Instead, our focus is on nonhuman primates and humans. Many specializations emerged at various times during primate evolution, including a dramatically enhanced reliance on vision and, in haplorhine primates, foveal vision [22].

We devote most of our attention to direct amygdala–PFC interactions, on the assumption that monosynaptic interactions are crucial for understanding the role these two regions play in behavior. Indirect, polysynaptic interactions are also important, of course; and, in any event, many relevant studies, such as functional connectivity analysis based on resting state fMRI, cannot distinguish direct from indirect influences.

## Anatomy

The anatomical evidence we will review is largely from nonhuman primates, in which experimental approaches can demonstrate connections directly. As mentioned, connectivity evidence in humans is most often based on temporal covariance of signals measured with fMRI or electrophysiology. Structural MRI applying diffusion tensor imaging also has been used in both humans and nonhuman primates to study white matter connectivity, but given current technical limitations, these data are best understood in relation to anatomical tracer studies, particularly for understanding connections passing through regions with crossing fiber tracts [23], as occurs in the region of the uncinate fasciculus as it enters the frontal lobe [24, 25].

**Fig. 1:**
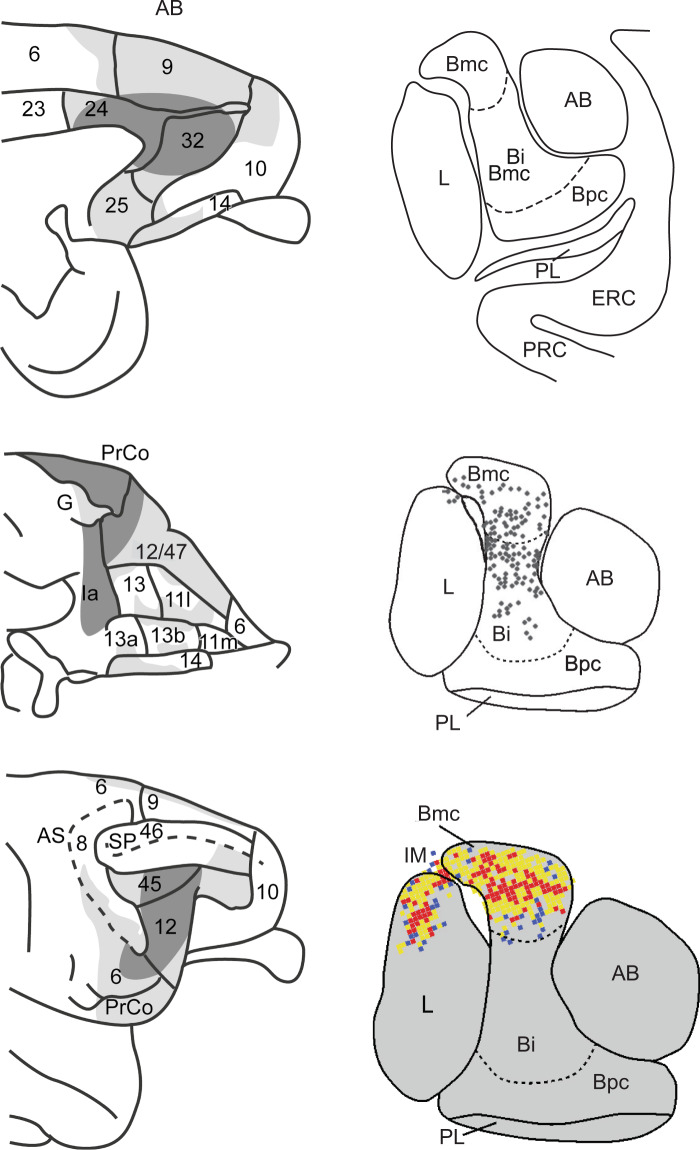
Anatomical connections of the amygdala and prefrontal cortex in macaque monkeys. Left. Schematic summary of macaque medial (top), orbital (middle) and lateral (bottom) frontal cortex regions in receipt of direct projections from the amygdala. Summary is based on anterograde tracers injected into the amygdala. The darker the shading, the greater the density of the terminal labeling. Numerals indicate cytoarchitectonic subdivisions. AS arcuate sulcus, SP principal sulcus, PrCo precentral opercular areas, G gustatory cortex, Ia agranular insular cortex. Adapted from [26]. Right. Coronal sections at the level of the mid-amygdala showing the typical layout of amygdala nuclei and the close proximity to neighboring entorhinal and perirhinal cortex (top), location of cells giving rise to projections to area 45 of VLPFC (middle), and locations of cortico-amygdala terminals received from area 45 (bottom). Adapted from Gerbella et al. [34, 234]. AB accessory basal nucleus, Bi basal nucleus intermediate division, Bmc basal nucleus magnocellular division, Bpc basal nucleus parvocellular division, ERC entorhinal cortex, IM intercalated masses, L lateral nucleus, PL paralaminar nucleus, PRC perirhinal cortex. Central and medial nuclei of the amygdala are not illustrated.

In primates, the amygdala gives rise to widespread projections to medial, orbital and lateral portions of the PFC, as illustrated in Fig. 1. However, the amygdala is not directly connected to all parts of the PFC and, within the prefrontal subregions that receive direct amygdala projections, the terminals are not uniformly distributed. This figure comes from anterograde tracer experiments conducted by Aggleton et al. [26]., which generally agree with other anterograde fiber tracing studies [27, 28] and those based on retrograde tracers [29–31]. There is one notable discrepancy in this literature that becomes important below. Aggleton et al. (Fig. 1), like Price and Drevets [18], concluded that the amygdala projects only sparsely to a central part of orbital area 13, designated area 13 m by Carmichael and Price [32]. In contrast, Saleem et al. [33]. reported a robust projection from the amygdala to this area (see also [28]). Perhaps the single case illustrated by Saleem et al. just happened to capture a particularly dense and highly localized amygdala termination zone.

In macaques, PFC projections arise mainly from the basal nucleus of the amygdala, especially its intermediate and magnocellular parts, and terminate exclusively in the ipsilateral hemisphere. Compared to these projections, meager PFC projections arise from the accessory basal and the lateral nuclei of the amygdala. As noted by Aggleton et al. [26], this contrasts with the amygdala’s connections with inferior temporal cortex (ITC), which has dense connections with both the lateral and basal nuclei.

For the most part, these amygdala-to-PFC projections are reciprocated, although the precise fields of origin in the PFC and termination within the amygdala may differ. For example, although both intermediate and magnocellular parts of the basal nucleus give rise to projections to area 45, a part of the ventrolateral PFC (VLPFC), the return projection terminates almost exclusively in the magnocellular basal nucleus. Furthermore, cortico-amygdala projections from area 45 terminate densely in the dorsal part of the lateral nucleus, which reciprocate that input weakly, if at all [34]. Unlike most other PFC subregions that receive amygdala projections, it appears that the anterior cingulate cortex (ACC)—areas 24, 25, and 32—sends more dense projections to the amygdala than it receives from it [28]. The functional significance of this relative weighting of projections is not understood, but might be elucidated with pathway-specific manipulations in nonhuman primates.

Another aspect of anatomy that deserves comment is the close relationship of both the amygdala and ventral and medial regions of the PFC to the hypothalamus [35–37], midbrain [38] and other areas [39] that, together with the anterior insular cortex, are implicated in interoception. These brain structures signal physiological arousal as well as other state variables such as hydration and temperature [40, 41]. Interactions with the hypothalamus mediate at least some of the sensory influences on autonomic and neuroendocrine systems. One intriguing idea [42] is that visceromotor signals to the hypothalamus and related areas not only maintain homeostasis, but also serve to predict interoceptive signals that are expected to arise as consequences of those allostatic visceral changes. If so, abnormalities in interoceptive predictions, like abnormalities in visceromotor outputs, could lead to autonomic dysregulation, which features prominently in mental illness. Notably, the PFC regions most strongly implicated in the pathophysiology of depression, such as the pregenual and subgenual ACC [18], are part of the visceromotor circuitry.

In humans, structural connectivity measured with diffusion-weighted MRI and probabilistic tractography, and functional connectivity measured with fMRI at rest or during tasks agrees, generally, with the connectivity demonstrated in macaques. There is evidence for connectivity between amygdala and medial, lateral (middle and inferior frontal gyrus) and orbitofrontal/ventromedial parts of the PFC [43, 44]. Notwithstanding the challenges in resolving nuclei within the amygdala with MRI, studies have attempted finer-grained parcellations, most commonly distinguishing basolateral, centromedial, and superficial clusters of nuclei, and have provided evidence for distinct patterns of cortical connectivity across these subdivisions [45, 46], or distinct relationships with resting-state networks that include PFC subregions [47]. In addition, there is evidence for projections between amygdala and frontopolar regions in humans for which there are no macaque homologs [48]. While studies in humans lack the anatomical precision of experimental neuroanatomy in macaques, they can help to validate the clinical relevance of data from these model species. As one example, a recent meta-analysis of 46 fMRI studies in patients with internalizing psychopathologies (e.g., depression, anxiety, post-traumatic stress disorder) or risk factors for these disorders found consistent differences between the patients and healthy controls in resting-state functional connectivity between amygdala and two adjacent regions within medial PFC: subgenual and pregenual ACC [49].

## Amygdala–PFC interactions in foraging

Evidence suggests that the amygdala interacts with select portions of the PFC to guide foraging for nutrients, i.e., the process of exploring for and exploiting specific foods, fluids, and essential vitamins and minerals. Here we review physiological and neuropsychological evidence relating to a subset of behaviors relevant to foraging in macaques (i.e., discovering, evaluating, and choosing between food-predictive cues) and bring in findings from humans when they are available. We note that research on foraging in humans addresses a broader portfolio of behaviors (such as temporal discounting, effort costs, and risk assessment) than will be reviewed here.

As indicated earlier, primates depend to a considerable extent on vision for finding and choosing among food items. The visual regions in the ITC and adjacent perirhinal cortex (PRC), a multimodal area dominated by vision in primates, project directly to the VLPFC (areas 12/47) and to the granular parts of the orbital PFC (area 11 and parts of area 13), more commonly known as the orbitofrontal cortex (OFC) [33, 50, 51]. Thus, nonspatial visual influences on foraging, such as information about colors, shapes, and visual textures, are likely to be mediated by these two parts of the PFC: VLPFC and OFC [52]. As shown in Fig. 1, these same PFC subregions receive inputs from the basolateral amygdala, suggesting that the amygdala–PFC projections are in a position to influence visual foraging. Finally, there are robust reciprocal connections between the basolateral amygdala and both the ITC and PRC [53–56] and evidence that projections from these temporal visual areas overlap with OFC projections to the amygdala, suggesting close interaction among these three cortical regions [57]. These connections are of interest because both the PFC [58–60] and the amygdala [61–64] have been proposed to modulate visual attention via their projections to ITC and PRC.

Murray et al. [65]. have suggested that the granular OFC, which emerged in early primates or perhaps in the tree shrew–primate common ancestor (see Preuss and Wise, this volume), provided a specific adaptive advantage during primate evolution: an enhanced ability to detect and to identify nutrients in dim light, especially in the cluttered, fine-branch niche that early primates exploited. Within the granular OFC, visual inputs from ITC and PRC converge with gustatory, olfactory and visceral sensations from the agranular OFC to establish cortical representations unique to primates [66] (see [67] for review). Together with another granular part of the PFC, the frontal eye field, visual inputs to the granular OFC improved the ability to search for and orient attention toward valuable items, both in peripersonal space and at a distance. Because the specializations of early primates were the starting point from which all modern primates descend, the function of the granular OFC endowed descendant species with functions that distinguished the granular OFC from the OFC of other mammals. Based on associative predictions that depended on vision and the control of visual attention, their new PFC subregions empowered early primates to forage “visually” for foods they could see only poorly, if at all, and modern functions of the granular OFC probably derive from that origin.

This idea highlights three fundamental aspects of foraging in primates: learning about visual cues that predict nutrients and predict the properties of hidden or poorly illuminated food items; estimating the current value of the predicted nutrients; and choosing among foraging options. Evidence suggests that amygdala–PFC interactions contribute to all three.

### Nutrient coding in OFC

Nutrient coding provides the foundation for learning, valuation, and choice among foods. A wealth of data point to a role for the OFC, a key part of the primate PFC, in representing the sensory properties and current subjective value of nutrients that are immediately available in the environment. For example, when monkeys gaze at an image on a monitor screen, neurons in OFC are active in relation to the anticipated fluid linked to that image. In fact, OFC neuronal activity reflects these properties not only when monkeys anticipate rewards but also when they receive rewards. OFC neuronal activity encodes several features of the nutrients that have been associated with those images, including the amount of fluid, the type of fluid (flavor), and its subjective value [68]. Taste-responsive neurons have also been found in the adjacent VLPFC (area 12/47), especially the orbital portion of area 12 (area 12o), as have neurons responsive to fat [69]. Accordingly, both OFC and VLPFC probably contribute to signaling the taste and texture of nutrients. Functional MRI studies using multivariate pattern analysis have shown that OFC activity in humans reflects identity-specific signals, suggesting OFC encodes odor identity. In contrast, signals reflecting the contextual (monetary) value associated with these odors were detectable in adjacent orbital and medial parts of the PFC (termed VMPFC) [70], as was an identity-general value signal that was linked to amygdala–VMPFC coupling [71]. Specific tastes are likewise represented in the OFC [72].

Consistent with these anatomical and physiological findings, evidence indicates that the granular OFC—the part specific to primates—is necessary for linking arbitrary visual cues and objects with unseen resources, typically foods, via learning. It is possible, but not yet established experimentally, that the visual properties of food items play an important role in these functions, in addition to the more obvious sensory correlates of biological value, such as gustatory, olfactory, and textural features of foods. In this way, arbitrary visual cues may indicate the presence of specific foodstuffs, and thereby predict them, including their visual features. Importantly, this prediction, or expectation, includes information not only regarding the sensory properties of nutrients, such as their visual appearance and taste, but their current desirability, as explained below. In keeping with this notion, humans with focal lesions affecting OFC can visually identify complex objects without difficulty, but show impairment in assessing the holistic desirability (i.e., monetary value) of such objects [73]. FMRI studies in humans have shown that patterns of activity in medial and lateral OFC reflect the subjective value (and nutritional composition) of foods presented as photographs [74–76]. One study, which employed a fMRI design based on repetition suppression, identified activations related to stimulus-food associations in the rostral OFC and food identity in the caudal OFC [77].

### Valuation

Of the learning, valuation, and choice aspects of foraging, the valuation aspect has been studied most. In practice, it is difficult to isolate the neural bases of choice from the other aspects of foraging that influence it. Accordingly, rather than discussing choice separately, we consider choice together with each of the other aspects.

Studies using the devaluation task have isolated a role for amygdala–OFC interactions in updating food value and linking that value to the visual or other stimuli that predict the availability of that food. In this task, monkeys are allowed to choose between pairs of objects, and each object of the pair predicts a different food reward, something each monkey had learned well before the main experiment. The key experimental manipulation is selective satiation; animals are allowed to eat one type of food until they reach satiety, i.e., the point at which the animal does not want any more of that food, indicating that its subjective value has dropped to zero (or perhaps below). Probe tests conducted after selective satiation reveal the ability of monkeys to link objects with the current, updated values of the foods. Importantly, each choice provides access to some food—either the devalued food or the nondevalued food.

**Fig. 2:**
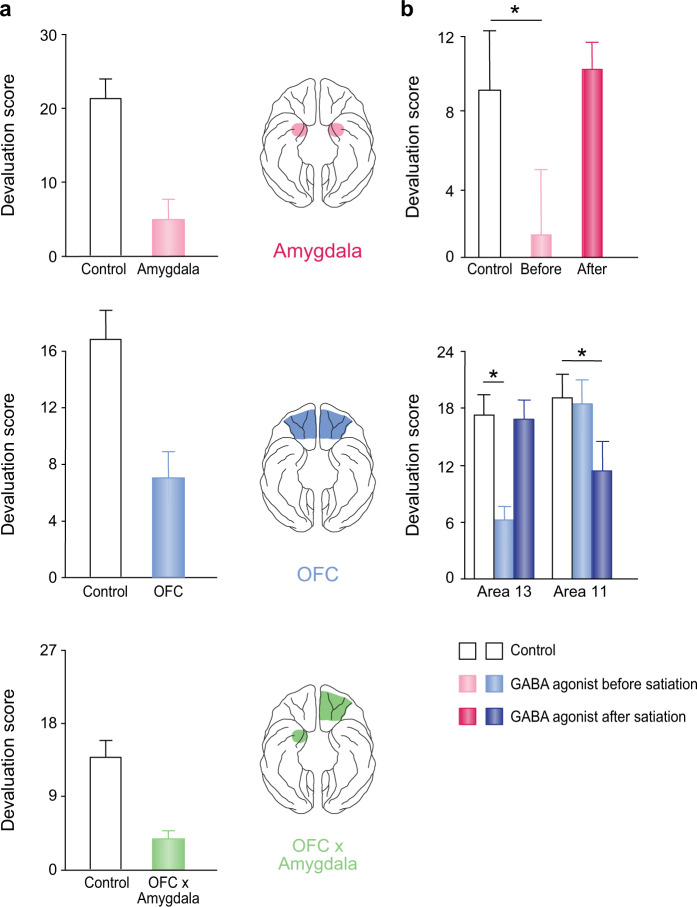
Selected studies revealing the neural underpinnings of performance on devaluation tasks in macaques. **a** Effects of bilaterally symmetrical or crossed surgical lesions of the OFC and amygdala on the devaluation task. “Devaluation score” reflects the extent to which object choices on the probe tests conducted after prefeeding differ from the baseline condition (no prefeeding). Higher devaluation scores reflect a greater proportion of adaptive choices after a change in food value. Data from [79, 81, 96]. **b** Effects of bilaterally symmetrical infusions of GABA agonists into either the basolateral amygdala (top) or different sectors of OFC (bottom). Infusions were given either before or after the selective satiation phase. GABA agonist infusions before satiation produced inactivation during both the satiation and object choice phases, whereas GABA agonist infusions after satiation produced inactivation during the object choice phase only. Scores of control groups vary across studies due to differences in methods. Data from [98, 99].

Intact monkeys spontaneously shift their choices away from the objects overlying the devalued food, as evidenced by their robust devaluation scores (Fig. 2a). This indicates that monkeys can estimate the current value of the foods that will result from their object choices. Scores are greatly reduced in monkeys with either bilateral lesions of the amygdala [78–80] or the OFC [80–82] (Fig. 2a, top and middle). By contrast, bilateral lesions of the VLPFC [82, 83] or hippocampus [84] have no effect. Control conditions rule out interpretations of these results based on motivation, basic food preferences, visual perceptual abilities, and satiety mechanisms. When given a direct, visual choice between sated and nonsated foods, the monkeys with either amygdala or OFC lesions choose the higher-value, nonsated food. Thus, the impairment caused by the lesions appears to be specific to linking objects with current food value or some associate of that value.

**Fig. 3:**
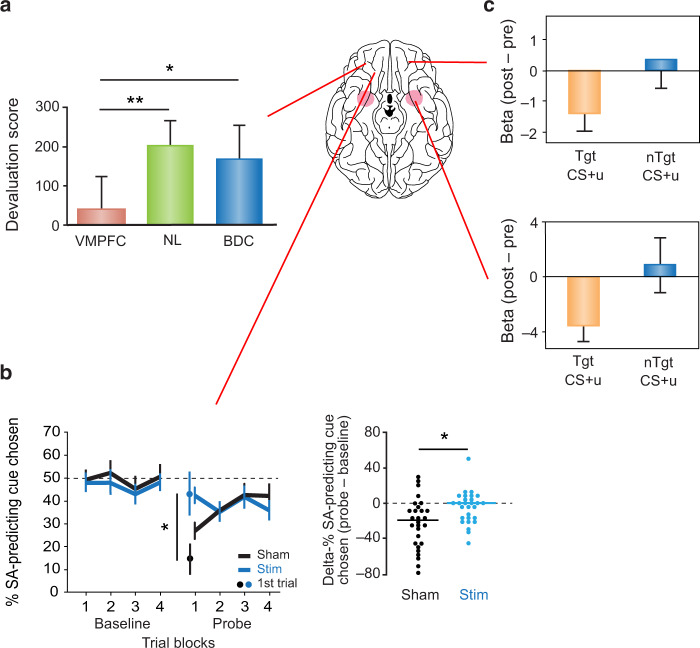
Selected studies revealing the neural underpinnings of performance on devaluation tasks in humans. **a** Effects of damage to the orbital and medial sectors of PFC (VMPFC) on the devaluation task in humans, relative to healthy participants (NL) and brain-damaged controls (BDC). Devaluation score shows change from baseline (presate-postsate responses to the image predicting the sated food). Data from [85]. **b** Effects on the devaluation task of continuous theta-burst magnetic stimulation (cTMS) applied to the lateral frontal cortex intended to disrupt the orbital frontal cortex network. Lower scores of Sham group indicate reduction in choice of the cue predicting the sated food. Data from [86]. **c** In an fMRI version of the devaluation task, neural responses elicited by the target CS (Tgt CS + u)—the visual stimulus that had been paired with the devalued odor—declined from pre- to post-satiety, whereas the nontarget CS (nTgt + u) activity was unchanged. OFC (top) and amygdala (bottom) signal change is plotted as contrasts of parameter estimates (betas) for both target and nontarget CS, after adjusting for CS– baselines. Data from [92].

There is also evidence for an effect of OFC damage on the devaluation task in humans. In one study, participants learned that each of two complex images predicted a different food. Then, just as in the macaque studies, participants were sated on one of the two foods and subsequently allowed to choose between the visual images to obtain yet more food. Reber et al. [85] found that patients with damage to the VMPFC continued to choose the image associated with the devalued (sated) food (Fig. 3a), indicating a failure to shift their choices to the image linked to the nondevalued food. In contrast, healthy control participants made this shift consistently. These findings resemble closely those in macaques with bilateral OFC lesions. The patients rated the hedonic value of the foods normally, and they reported a marked decrease in the pleasantness of the sated food, yet they failed to reduce choices that yielded a food item they no longer deemed valuable. Both macaques with OFC lesions and humans with damage to VMPFC exhibited a disconnection between knowledge and action, also known as goal neglect. In each case, the subjects appeared to know the current, relative value of the two foods but were unable to choose the images or objects that gained them the nonsated, temporarily preferred food item.

Another recent study using a different method provides evidence that it is specifically OFC (rather than the broader VMPFC region) that is important in devaluation task performance in humans, as in macaques. This study made use of continuous theta-burst stimulation (cTBS) applied to lateral PFC to disrupt indirectly the functional connectivity of the OFC network. Using a version of a devaluation task in which visual images predicted odors of sweet and savory foods, it was found that participants who underwent cTBS, unlike controls, continued to choose cues that predicted devalued odors, failing to shift to the item that, for the time being, had the higher value (Fig. 3b). Importantly, their ratings for the value of each food odor were unaffected by the stimulation. This study points to OFC, possibly in combination with lateral PFC, as an essential part of the circuitry mediating devaluation effects in humans [86].

In addition, fMRI studies in humans implicate the OFC and VMPFC in the subjective valuation of outcomes associated with visual stimuli, such as money, food, and odors [71, 87, 88]. Related experiments with satiety manipulations have shown a reduction in the BOLD signal in the OFC in concert with a drop in subjective pleasure or value due to satiation [89–91]. Likewise, after selective satiety had been established, BOLD signals related to visual cues predicting sated food odors were reduced in both the amygdala and OFC (Fig. 3c) [92].

In keeping with the lesion findings, physiological studies in macaques have revealed that the activity of single neurons in VLPFC (area 12o) and OFC (areas 11, 13, and 14) reflects satiety states [93, 94]. Although early work placed an emphasis on the reduction in firing rate that accompanied satiety [93], one study found that roughly equal numbers of neurons showed increases and decreases in firing rate [94].

Given the results from amygdala and OFC lesions, it seems likely that their functional interaction is essential to dynamic revaluations, as well. To test this possibility directly, monkeys received crossed-disconnection lesions of the amygdala and OFC (i.e., removal of the amygdala in one hemisphere and of the OFC in the other), which prevents their intrahemispheric interaction. Monkeys with these lesions, like those with bilaterally symmetrical lesions of the amygdala or OFC, were impaired on the devaluation task [95, 96]. In addition, a separate study replicated the crossed-disconnection lesion effect using a task in which actions, rather than objects, were linked to foods [97]. These studies demonstrate that the amygdala must functionally interact with the OFC within a hemisphere to guide adaptive choices (Fig. 2a, bottom).

Although the lesion method establishes that the amygdala and OFC must functionally interact to mediate the devaluation effects, this work does not show how the devaluation effects occur. There are at least two explanations of how performance on the devaluation task could be disrupted by the crossed-disconnection lesions. First, if value is represented in the OFC, perhaps the amygdala–OFC interaction mediates a value-updating function during selective satiation. A failure to register the change in food value would ultimately lead to the impairment observed on the devaluation task. Alternatively, perhaps the value-updating process remains intact, but there is an inability to retrieve the updated value at the time of object choice, based on the visual properties of the object associated with that food. These two possibilities—an updating impairment versus a retrieval impairment—have been evaluated by performing pharmacological manipulations at different time points during the devaluation task. Temporary inactivation of either the OFC or amygdala during the selective satiation procedure probes the ability to update value; whereas during the choice phase, which occurs after selective satiation, inactivation probes retrieval.

These studies have produced three findings:

1. Inactivation of the basolateral amygdala during the selective satiation phase, but not later during the choice test phase, disrupts performance on the devaluation task (Fig. 2b, top) [98]. Thus, the amygdala must be active during selective satiation to register the change in food value. Once the amygdala has participated in this value-updating function, it is no longer required for making the best choice.
2. Reversible inactivation of granular OFC area 13—the caudal part of OFC—yields an identical pattern of results [99], suggesting that the amygdala and area 13 work together to perform the value updating (Fig. 2b, bottom). This finding explains why the discrepancy in anatomical findings, noted above, is important. The experiment just cited centered the inactivation on area 13 m, and the finding by Saleem et al. [33]. of a robust amygdala–cortical projection to area 13 m is more consistent with these findings.
3. Inactivation of granular OFC area 11—the rostral part of OFC—has its effect not during the selective satiation phase but during the choice phase instead (Fig. 2b, bottom) [99]. It appears that if area 11 was active during value updating, it is necessary for linking objects with updated value at the time of choice, i.e., value retrieval rather than value updating. Thus, OFC area 11 is necessary for goal selection, which requires identifying which of two choice objects is linked to the higher value food.

Related information regarding amygdala interaction with the OFC comes from a neurophysiological recording study in macaques in which neurons in OFC were recorded both before and after bilateral excitotoxic lesions of the amygdala [100]. This study confirmed previous findings that a substantial proportion of OFC neurons encode the expected magnitude of the reward. Removing amygdala inputs to the OFC significantly reduced, but did not abolish, the encoding of reward value in the OFC during the evaluation phase, while monkeys viewed the images available for choice, and around the time of reward delivery. These findings suggest a role for the amygdala in the active maintenance of the neural representations of learned associations between visual stimuli and current valuations. Importantly, amygdala lesions have this effect in OFC but not in the medial PFC.

### Learning

Selective satiety is one factor influencing foraging behavior, thereby driving dietary diversity. As more of a food is consumed, the drop in reward value makes it more likely that an individual will select other food options. Although studies using selective satiation have identified a role for amygdala–PFC interactions in foraging choices based on familiar stimulus-reward associations and multiple food outcomes, they do not address the underlying stimulus-reward learning.

Work in rodents, much of it based on Pavlovian conditioning, has identified roles for agranular OFC and basolateral amygdala in learning from reward and punishment [101–104]. A detailed discussion of this work is beyond the scope of this review, but we highlight some selected points that inform our main themes (for review see [105]). Work in appetitive conditioning in rodents is addressed later in this section, whereas some key work in aversive conditioning in rodents is discussed in the next section.

In appetitive conditioning, the central nucleus of the amygdala (Ce) is important for processing the general motivational value of stimuli, whereas the basolateral group of amygdala nuclei (BLA) is important for processing specific-sensory properties of the unconditioned stimulus, which is often a food item [106]. A stimulus that predicts food will elicit learned approach to a food source (also known as goal tracking), along with behaviors favorable to ingesting that food (e.g., chewing, biting, salivation). Furthermore, depending on the setting, animals will approach to the conditioned stimulus itself (also known as autoshaping and sign tracking). In sum, together, the BLA and agranular OFC acquire stimulus-outcome (S–O) associations. The nature of this learning is that the behavior elicited by the conditioned stimulus is obligatory. The specific roles of the BLA and OFC are a matter of debate, but many authorities have suggested that the amygdala is important for acquiring reward representations, whereas the OFC is more important for using this information to generate expectations that guide foraging choices [104, 107–110]. These findings extend the conclusions from primates reviewed above. Both the granular OFC of primates and the agranular OFC of rodents appear to represent the sensory properties of foods and link these representations to stimuli that predict these properties.

In other studies carried out in rodents, Wassum et al. have found that the interaction between the agranular OFC and BLA is essential during both the encoding and retrieval of reward value, and the interaction during retrieval is only necessary when the reward is unobservable, i.e., when a representation of the reward needs to guide behavior. Corticofugal connections and inputs to the OFC play different roles. Whereas projections from BLA to OFC are essential for a stimulus to elicit specific reward expectations [111], the reciprocal, corticofugal projections, from OFC to BLA, are essential for encoding positive changes in the value of food associated with a tone, and for retrieving value representations to support bar presses that produce the same reward [112]. These effects in BLA are mediated by NMDA receptor-dependent synaptic plasticity [113], and are doubly dissociable, with projections from lateral OFC to BLA being important for encoding, and projections from medial OFC to BLA being important for retrieval [112]. Furthermore, optogenetic stimulation of OFC-to-BLA projections induced positive value changes, and this stimulation was sufficient to yield behavioral changes that otherwise would not have occurred [112]. These results have implications for amygdala–PFC interactions in primates. Because the agranular parts of OFC are thought to be homologous with those in primates, it will be important to establish whether the agranular OFC in primates plays a similar role as in rodents, with a related distinction between corticofugal and amygdala–cortical projections. Future research could also explore whether granular PFC areas implicated in valuation in primates, such as the VLPFC and granular OFC, interact with the amygdala in the ways suggested by the rodent studies of agranular OFC.

In primates, stimulus-reward learning with single outcomes has been studied extensively using associative learning tasks. The amygdala, OFC, and VLPFC have been shown to be important for aspects of such learning in monkeys and humans. There are two important factors to consider. First, although there is a sizeable literature in nonhuman primates addressing the neural bases for stimulus-reward learning, it is now clear that studies using excitotoxic lesions provide a dramatically different picture of the functions of these parts of the PFC, and of the amygdala, compared to studies using aspiration lesions [81, 114]. This is because aspiration lesions, unlike excitotoxic lesions, disrupt fibers of passage, a problem exacerbated by the complex white matter tracts passing nearby the amygdala and caudal OFC [115]. Accordingly, we focus on findings from studies using excitotoxic lesions and other, similarly selective manipulations, where available. Human lesion studies are unavoidably much less selective, and thus are best interpreted in the context of the more selective lesions possible in nonhuman primates [116–118] and clues about localization from fMRI studies. Second, the nature of what is learned in stimulus-reward learning likely differs in rodents and primates. Both encompass S–O learning related to the sensory properties of the predicted food outcomes, but additional representations might be specific to primates, such as those for the visual properties of a food reward independent of its motivational value (i.e., reward-free representations of what food items look like).

In primates, reward learning involves both the OFC and VLPFC (area 12/47). To our knowledge, there are no studies that directly test the idea that the amygdala must functionally interact with the PFC in stimulus-reward learning. However, there is evidence that the PFC [83, 119, 120] and amygdala [121, 122] each make a critical contribution to such learning in nonhuman primates and humans. A substantial lesion literature shows that damage to VLPFC impairs flexible stimulus-reward learning, i.e., when the reward is only probabilistically related to the stimulus, or when contingencies change, such as in reversal learning, or in so-called “bandit” tasks in which the probability of reward assigned to different images fluctuates over time. Such tasks require the linkage of a given reward outcome with a specific stimulus occurring in a stream of temporally adjacent stimulus-outcome events, referred to as the credit-assignment problem [123]. Computational modeling shows that the influence of a given instance of rewarding feedback “blurs” its influence, and so affects the likelihood not only of choosing the specific stimulus that preceded that feedback (trial n), but also the stimuli presented in temporally adjacent trials (n-1, n+1, n-2, n+2,…). Macaque, marmoset, and human lesion studies implicate the ventral frontal lobe, and specifically VLPFC, in credit assignment (macaques:[83, 124] marmosets: [125] humans: [126]). A part of VLPFC, area 12o, is a key region in probabilistic stimulus-reward learning; [127] BOLD signals in this region predict adaptive behavioral adjustments consistent with contingent learning (i.e., optimal credit assignment) [128]. The latter study also reported greater connectivity between the amygdala and VLPFC when reward was more informative. Neurons in more lateral portions of the macaque VLPFC encode valuable objects—images associated with a large magnitude of reward—in long-term memory [129], and long-term value-related fMRI signals were found in the VLPFC as well as in other anatomically connected regions such as the ITC and the amygdala [130]. Finally, monoamine levels in the VLPFC are also related to learning rates [131].

In humans, damage to ventromedial frontal regions, including medial OFC and VMPFC (as well as fibers of passage) disrupts deterministic and probabilistic stimulus-reward reversal learning [132–134]. This may not be due to impaired learning (i.e., credit assignment), but rather to a deficit in comparing option values under challenging, dynamic conditions [126], although more definitive evidence is needed to support this claim in humans.

BLA damage in both humans and nonhuman primates affects reversal learning as well, as it does in rodents, but this literature is more difficult to summarize. One synthesis argues that the BLA tracks past outcomes and compares that history to the current outcome, flagging unexpected deviations that signal a need to change behavior [135]. This idea has obvious relevance to adjusting to dynamic shifts in reward contingencies during associative learning, and it accords with the observation that animals with amygdala lesions sometimes learn reversals faster than intact control subjects, especially early in learning. This finding has been attributed to overall weaker learning, which makes animals more likely to choose the newly-rewarded option because the reward association with the previously best choice is weaker initially [136].

In sum, there is abundant evidence that both amygdala and PFC contribute to learning the value of visual objects through reward feedback, especially in dynamic or probabilistic settings, and some evidence that these regions interact to support such learning. VLPFC (perhaps area 12o specifically) may be especially important for foraging choices in the face of multiple competing visual cues or irrelevant feedback [137–139] or when generalizing prior experience to novel objects [140], a capacity related to analogical reasoning. In contrast, OFC is more important when the value of nutrient outcomes changes. In each case, the PFC–amygdala interactions allow recently learned information to modulate amygdala output, achieving the level of motivation appropriate for the new situation.

## Amygdala–PFC interactions in predator avoidance

Like foraging, engaging in adaptive responses under threat is another crucial aspect of Darwinian fitness. Maladaptive responses present as either underresponding to immediate threat, leading to vulnerability to predation, or overresponding, leading to a reduced ability to engage in essential activities. Because excessive attention to threat characterizes individuals with anxiety disorders [141, 142], identifying the brain areas involved in regulating responses to threat holds some clinical relevance [17, 143]. Some aspects of predator avoidance are innate. For example, rats raised in a laboratory that are naive to predator odors display avoidance and defensive behaviors when first exposed to the urine of predators (e.g., canids and felids), but not when exposed to the urine of nonpredators (e.g., ungulates) or conspecifics [144]. Similarly, snake-naive macaques raised in the laboratory show a range of defensive and withdrawal responses when confronted with a snake, but not when confronted with novel, neutral objects [7]. These behaviors rely mainly on subcortical circuits that include the amygdala. For example, studies using assays that involve exposure of rodents to predator urine, predator body odor, or TMT—a compound found in fox feces—have revealed a circuit comprised of the medial nucleus of the amygdala, ventromedial hypothalamus, the premammillary nucleus, and the periaqueductal gray (PAG). In addition, parts of the BLA are active when visual or auditory predator cues are presented. Anatomically, it appears there are parallel circuits running through these structures that mediate defensive responses to learned stimuli, predators, and conspecifics, at least in rodents [145, 146]. These findings have implications for treatment of social anxiety disorders and small-animal phobias, where selective manipulation of anatomical pathways could prove beneficial.

Neuroimaging studies in humans and neuropsychological studies in monkeys implicate similar sets of brain regions in the adaptive response to predator threat, including the amygdala, hippocampus, bed nucleus of the stria terminalis, and the PAG [147–151]. Many of the amygdala outputs are to subcortical structures that mediate defensive responses [152]. Human studies, which use photos of threatening stimuli, have sometimes been criticized for a lack of ecological validity. However, an fMRI experiment in which human participants believed a real tarantula was nearer or farther from their foot while they lay in the scanner showed increased activity in the amygdala and also dorsal ACC in addition to many of the threat-sensitive regions listed above, in proportion to the proximity of the spider [153].

One assay for predator avoidance in macaques is the snake test. Approaches to obtain a piece of food are pitted against defensive responses engendered by either a fake or real snake or, on other trials, a fake spider. The dependent measure is food-retrieval latency. Many investigators have reported that selective, excitotoxic lesions of the amygdala markedly reduce monkey’s reactions to snakes and spiders [154–158]. Whereas monkeys with complete excitotoxic lesions of OFC areas 11, 13, and 14 were no different from controls [81], monkeys with subtotal lesions of OFC showed heightened defensive and reduced approach responses in the presence of the threatening stimuli, accompanied by longer latencies to retrieve a food reward [159]. In addition, monkeys with medial OFC lesions displayed a greater tendency to express defensive responses in the absence of threat. Overall, the data indicate that, when intact and functional, both the medial and lateral OFC contribute to the attenuation of defensive responses. Notably, these findings, obtained with selective, excitotoxic lesions of OFC, agree with work in marmosets showing that excitotoxic lesions of anterior lateral OFC (primarily area 11) and VLPFC (area 12) resulted in heightened defensive responses to a fake snake [160]. The overall pattern of results suggests a critical role for the OFC, and perhaps also the VLPFC, in adaptively attenuating defensive behaviors over repeated presentations of a threat stimulus in the absence of overt negative outcomes. These observations raise the possibility that dysfunction in one or more these PFC subregions could be the basis of the enhanced threat responses characteristic of anxiety disorders, such as phobias or PTSD.

Some models posit that a top-down inhibitory role of PFC on limbic and midbrain areas mediates this attenuation [161, 162]. The finding of heightened defensive responses following selective, excitotoxic OFC damage in macaques is consistent with these models. However, the idea that the PFC predominantly inhibits amygdala output is contradicted by neuroanatomical evidence from both rodents and macaques. Cortical projections to the amygdala, which are excitatory, terminate on both excitatory and inhibitory interneurons [163–165]. Thus, cortical input can both suppress and enhance amygdala outputs. A better understanding of this interplay will be crucial if the promise of so-called precision psychiatry in treating anxiety symptomatology is to be fulfilled.

Yet another way to achieve modulation of threat responses is via opposing influences from the PFC. For example, studies examining the neural substrates of aversive conditioning have found that rodent infralimbic cortex (ILC, homologous to the subgenual ACC in primates) is essential for suppressing threat responses and enhancing threat extinction. In contrast, rodent prelimbic cortex (PLC, homologous to the pregenual ACC in primates) is essential for enhancing threat behaviors. Both threat suppression and enhancement involve the amygdala [166, 167]. Thus, ILC and PLC, through interaction with the amygdala, permit bidirectional control of responses to threat (see [117] for related work in nonhuman primates). Intriguingly, ILC also exerts a similar influence on behavior in appetitive settings [168], suggesting an overarching role for PLC and ILC in biasing one type of association over another when competing or contradictory associations vie for control of behavior [65]. (A treatment of findings integrating PFC, amygdala, and hippocampal contributions to responding to threat is beyond the scope of this article; for review see Anderson and Floresco, 2020 and Kredlow et al., 2022 in this volume.)

Some studies in humans suggest functional specializations of the lateral and medial OFC in processing predator information. For example, a comparison of the neural responses of spider-phobic and nonphobic subjects to spider stimuli revealed decreased activation in the lateral OFC, which was normalized by cognitive behavioral therapy [169]. In the experiment mentioned above involving a real tarantula, the medial OFC exhibited greater activation as the tarantula became more distant, perhaps reflecting a regulatory effect [153].

Defensive responses to snake stimuli but not neutral stimuli can be conditioned through observational learning, indicating that defensive responses to snakes in macaques, although innate, are capable of being modified through experience [170–172]. The exact mechanisms by which the macaque OFC might contribute to learning about safety remain unknown. One possibility is that information about the absence of negative outcomes following their expectation may be integrated in brain areas such as the amygdala, PAG, etc. to downregulate behavioral and physiological responses to stimuli. This body of work may be relevant to optimizing the trade-off between exploring for potential reward opportunities and avoiding potential threats. For example, foraging games where humans seek monetary rewards but risk large losses also engage threat-response regions, including the amygdala and dorsal ACC [173, 174]. Consistent with a role for the amygdala in prioritizing threat, patients with selective amygdala damage show altered return to safety in an approach-avoidance conflict task [21].

## Amygdala–PFC interactions in social communication

In primates, foraging for food involves identifying visual cues that predict reward, often in rapidly changing contexts. Likewise, social communication involves a wealth of visual cues. In humans and nonhuman primates, both the amygdala and OFC play a role in the visual processing of faces and facial expressions of emotion, as well as processing emotionally significant stimuli more generally [175, 176]. Other PFC regions, including frontopolar regions that lack homologs in macaques, have also been implicated in the social regulation of human behavior via emotion-laden visual signals, potentially through interaction with the amygdala [48].

### Faces

The amygdala may have a general role in attending to faces and detecting their emotional signals [176, 177]. Functional MRI studies have revealed multiple “face patches” in the ITC in nonhuman primates and humans [178–180]: regions of cortex in which activations are greater for faces relative to objects. Patients with damage to the amygdala are deficient in identifying facial expressions of emotion [181], specifically fear expressions, and have altered viewing patterns of faces, spending less time than controls in looking at the eye region when seeking information about fear. Valuable insights have been acquired from the study of patients with Urbach-Wiethe disease, a rare developmental disorder characterized by selective bilateral damage to the amygdala. When one such patient was instructed to look at the eye region, her ability to identify fearful facial expressions was reinstated [182]. Thus, the amygdala may be important for directing gaze to informative regions of the face, such as the eyes, perhaps specifically for fear recognition. The amygdala has also been implicated in the rapid detection and attentional prioritizing of emotionally-relevant visual information more generally, although the evidence in support of this idea is mixed [64]. For example, emotional stimuli can reduce the attentional blink in rapid serial visual presentation paradigms, arguing that emotion boosts early attentional prioritization. Likewise, visual search tasks show some prioritization of emotional compared to neutral faces [183]. Amygdala lesion studies in humans have reported mixed results with such tasks, with some investigators reporting a loss of the emotional-attentional prioritization after such damage e.g., [64, 184], and others finding normal performance [185]. Variations in tasks, lesion laterality, acuity and extent may explain these discrepancies [176]. Finally, the amygdala also has a role in assessing the emotional content of other socially-specific sensory inputs, such as voice signals [186]. For example, acute amygdala damage following stroke is associated with specific deficits in detecting fear (but not other emotions) from language prosody [187].

Like humans, macaques presented with images of faces preferentially view the eye region [188]. Relative to intact monkeys, those with amygdala lesions spend less time viewing the eye region and more time viewing the mouth region [189]. A recent study found, in addition, that when monkeys with amygdala lesions were simultaneously presented with images of faces and objects, they made fewer first-looks to faces and spent less time viewing faces than did intact monkeys. Instead of directing eye movements toward socially relevant features, monkeys with amygdala lesions were biased toward features with increased low-level salience [190]. Likewise, physiological studies have identified neurons in the amygdala that signal face identity, facial expressions of emotion [191, 192], and the gaze of conspecifics [6]. Intracranial amygdala recordings in humans confirm many of these findings (reviewed in [193]).

The role of the PFC in these functions remains largely unknown. Electrophysiological recording studies in macaques viewing images of conspecifics report neurons sensitive to faces in both OFC and VLPFC [194, 195], findings mirrored by fMRI studies [196, 197]. Importantly, many of the face-sensitive neurons in PFC encode individual identity. In addition, some neurons in macaque OFC encode particular facial expressions (e.g., threat) and social categories (e.g., juveniles, females) [195]. Neurons in VLPFC are sensitive to whether visually perceived facial movements and vocalizations in video clips of vocalizing macaques “match”, suggesting a role for these neurons in integrating visual and auditory cues used in social communication [198]. Recent work examining ITC face patches has found a similar modulation of face-sensitive neurons by auditory cues to that observed in VLPFC [199]. Notably, this modulation was robust in face patch AF, located in the cortex along the banks and fundus of the superior temporal sulcus, but not patch AM, which is located more anteriorly and laterally within ITC. This finding suggests a possible segregation of function of these two face processing regions. Although there are robust direct projections from the amygdala to the rostral face patches (AM, AL), there appear to be weak projections from OFC and VLPFC to them [200]. Study of the connections of physiologically identified face patches is in the early stages, however, and the connections of only a subset of all face patches have been studied. More work investigating both PFC and amygdala connections with ITC face patches will no doubt be informative.

Patients with OFC lesions have at least a mild impairment in recognizing negative emotions [201], with some showing more severe deficits [202]. However, they direct their gaze to the relevant facial features in patterns similar to healthy people [203]. Thus, although the amygdala and OFC both seem to be critical for the recognition of emotion from visual face cues, the available evidence suggests they make different contributions.

### Beyond faces

More generally, neuropsychological research in humans points to a causal role for the amygdala and the medial PFC, especially the ACC, in social cognition [204, 205]. Patients with Urbach-Wiethe disease are profoundly impaired in their ability to learn from social information [206]. Similarly, patients with lesions that compromise substantial portions of both orbital and medial PFC are impaired in several aspects of social cognition [202, 207–213]. Deficits include contextually inappropriate social behavior, poor insight into social behavior, altered judgments about moral transgressions, altered recognition of voice and facial expressions of emotion, diminished empathy, and impaired mentalizing.

Research in nonhuman primates also provides evidence on the role of the amygdala and ACC in social cognition. Mirroring the findings in humans, monkeys with amygdala lesions are able to express social and emotional behaviors, but often do so in a context-inappropriate manner [214]. Amygdala neurons encode several features relevant to social interactions, many of which are described below. Importantly, single neurons in the amygdala can encode both social and nonsocial information [215, 216], suggesting that individual neurons, and the ensembles of which they are a part, provide yet another avenue for behavioral flexibility [175]. We discuss three areas of investigation: observational learning, prosocial tendencies as revealed by a reward-allocation task, and social interest and/or valuation.

For observational learning, investigators have devised tasks that require the interaction of two or more conspecifics [217, 218] or one macaque and one human [219]. These tasks require that the monkey observes the choices of the other agent (conspecific or human) while they take turns performing a task. In these studies, neurons in the dorsomedial [217] and lateral PFC [220] selectively encode which agent is performing the task, in addition to encoding other features such as the spatial position of the target and the conjunction of agent and target. (Cells in the dorsal premotor cortex have similar properties [219]). The distinction between self and other is thought to be essential for cooperative social interactions.

Another task evaluates the prosocial tendencies of macaques by measuring the amount of reward obtained by conspecifics [221]. In the social reward-allocation task (also known as the vicarious reinforcement task), two monkeys—an actor and a recipient—view cues presented on a video monitor. The actor monkey chooses between visual cues that signal delivery of juice to self, a conspecific other, both, or neither [221]. Importantly, the actor’s choices have no effect on his or her chance of obtaining a reward. For example, on some trials actors are offered a choice between cues signaling reward to the conspecific alone (Other), or to neither monkey (Neither). Under these conditions, monkeys show a prosocial tendency, making more Other than Neither choices. As a crucial control, monkeys do not make this choice if juice is delivered to a nonsocial entity, such as graduated cylinder. A series of studies using variations of this task have revealed that activity of neurons in the amygdala, OFC, and medial PFC (specifically the ACC) encode aspects of task performance [222, 223].

**Fig. 4:**
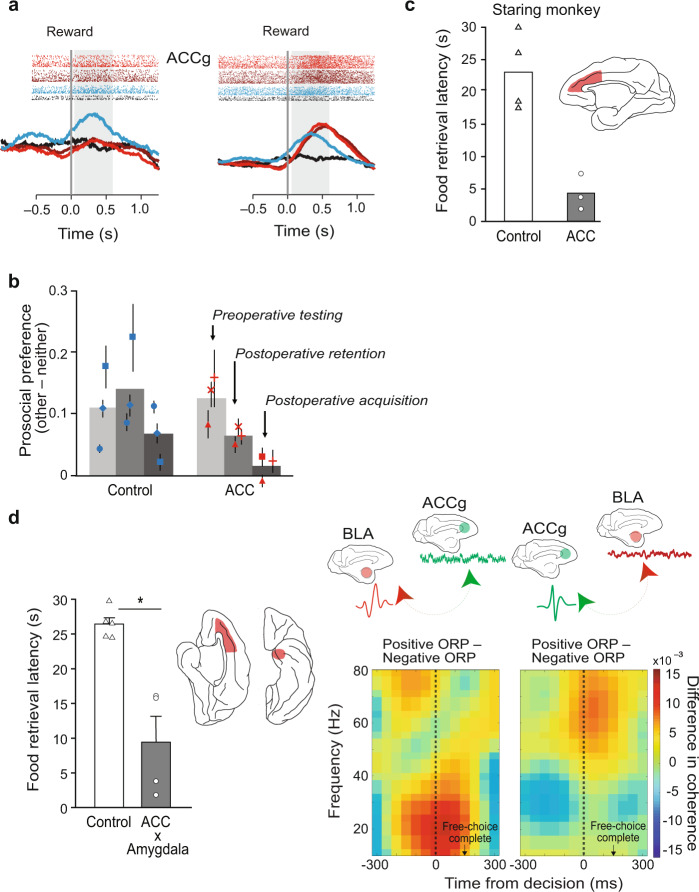
Selected studies illustrating the contributions of the ACC and amygdala to social other-regarding behaviors in macaques. **a** Neural correlates of choice during the reward allocation task in macaques. Plots show peri-stimulus time histograms and spike rasters for different conditions. Data are aligned to choice. Some neurons in the ACC gyrus encode reward to Other (blue) and others encode reward to Self (red and purple) as well as Other (blue). Data from [222]. **b** Effects of ACC (gyrus and sulcus) excitotoxic lesions on a social reward allocation task. Monkeys were given the opportunity to give rewards to Self, a conspecific (Other) or to no one (Neither). Prosocial tendencies are evidenced by the greater proportion of trials in which monkeys gave reward to Other relative to Neither. Data from [224]. **c** Effects of ACC (gyrus) aspiration lesions on a test of social interest. Macaques watched brief videos with social or nonsocial content. Dependent measure was the latency to retrieve food while videos were on display. Data are from the “Staring monkey” condition of [228]. **d** Left. Effects of crossed lesions of the amygdala and ACC on a test of social interest. Compare and contrast with (**c**). Data from [96]. Right. Oscillatory neuronal interactions between the basolateral amygdala and the ACC gyrus while monkeys expressed positive or negative other-regarding preference (ORP) in the social reward allocation task. Differences in spike–field coherence between the positive ORP (choosing Other over Neither) and the negative ORP (choosing Self over Both) exhibited frequency specific coordination as a function of the area that contributed spikes in the pair. Synchronization between the two nodes was enhanced for a positive ORP but suppressed for a negative ORP. Left panel: Differences in BLA_spike_–ACC_field_ coherence values between a positive ORP and a negative ORP over time. Frequency is aligned to the time of free-choice decision. Right panel: Difference in ACC_spike_–BLA_field_ coherence values between a positive ORP and a negative ORP over time and frequency. Data from [225].

During the performance of these tasks, amygdala neurons encoded reward magnitude on social decision trials, but did not carry information about the agent that received the reward [223]. OFC neurons signaled primarily reward to self. In contrast, the ACC contained three separate populations of neurons that signaled social outcomes: one that signaled rewards to self, another for rewards to a conspecific, and a third one with shared signaling of reward to self and the conspecific [222]. The ACC also contained neurons signaling reward to no one. Within the ACC, it appeared that the anterior cingulate gyrus, or pregenual cortex, was the region that could signal shared reward experience (Fig. 4a). Consistent with these findings, lesions of ACC that include the pregenual cortex disrupt macaques’ prosocial tendencies in this task (Fig. 4b) [224]. Whether the lesions disrupt the rewarding aspects of giving juice to conspecifics or, alternatively, the mapping of the visual cues to the different reward conditions, remains an open question. Nevertheless, taken together, the findings strongly implicate the ACC in learning about and representing other agents and their ability to obtain necessary resources. The fact that anthropoids such as macaques typically forage in groups is probably relevant to these findings.

The physiological recording studies mentioned above also investigated amygdala interactions with ACC during social decision making. This was done by analyzing spike-field coherence while monkeys performed a variant of the reward-allocation task. Spiking activity of individual cells in each area was related to the local field potential (LFP) oscillations in the other area [225]. The main finding was that there was enhanced neural synchrony between the amygdala and ACC during prosocial decision making (Fig. 4d). The timing of the coherence indicated it was unlikely to be responsible for generating the decision; instead, the authors suggested that the enhanced coherence might serve as a feedback mechanism that could be used to adjust future prosocial decisions.

As indicated earlier, macaques prefer to view images of conspecific faces over objects. Macaques also forego juice rewards to view images of female perinea, a secondary sexual characteristic of many anthropoid species, and the faces of dominant male monkeys [226, 227]. These findings suggest that social cues have intrinsic value, and neuropsychological studies investigated the neural bases of “social interest” or social valuation in this context. In one type of social valuation task, monkeys are allowed to obtain a piece of food from a tray while videos with social or nonsocial content play on a monitor located immediately behind the food. The dependent measure is food-retrieval latency. In this setting, unoperated control monkeys are, on average, slower to reach for food rewards when presented with videos with social compared to neutral, nonsocial content [228]. By contrast, monkeys with ACC lesions are quick to retrieve the food on trials with social videos, suggesting diminished value of social information (Fig. 4c).

**Fig. 5:**
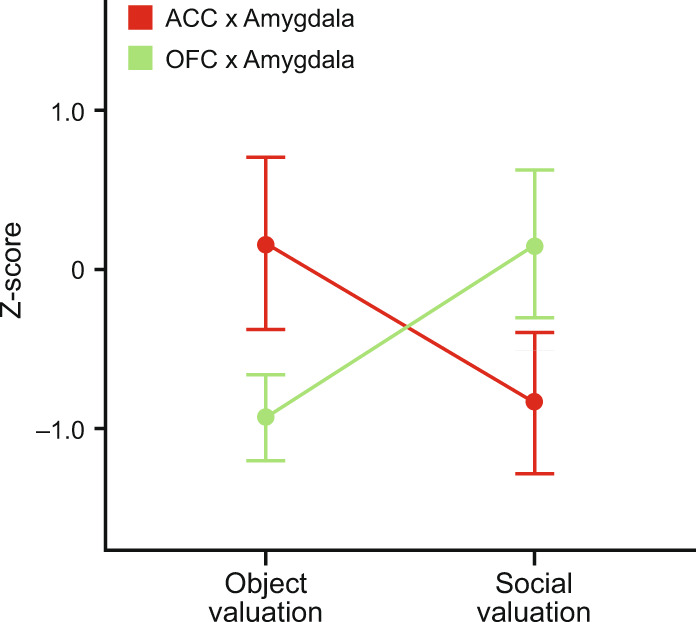
Double dissociation of function between groups with crossed lesions of the amygdala and either OFC or ACC. Monkeys with a surgical disconnection of the OFC and amygdala were impaired in object valuation but not social valuation. Monkeys with disconnection of the ACC and amygdala showed the converse result. The y-axis displays normalized *z*-scores, with error bars displaying the SEM. Data from [96].

More recently, functional amygdala–PFC interaction was investigated in the social-interest paradigm, and results were compared with those from the devaluation task, discussed earlier. One group of monkeys received crossed-disconnection lesions of the ACC and amygdala, and the other received crossed-disconnection lesions of the OFC and amygdala. Like monkeys with bilateral lesions of ACC, monkeys with crossed ACC–amygdala lesions showed significantly reduced food-retrieval latencies in the presence of videos of conspecifics, indicating reduced social valuation and/or interest relative to controls (Fig. 4d) [96]. Monkeys with crossed OFC–amygdala lesions did not differ from the controls on this task. The converse pattern of results was obtained on the devaluation task: monkeys with crossed OFC–amygdala, but not those with crossed ACC–amygdala lesions, displayed deficits on object choices following changes in food value. These findings indicate that both the ACC and OFC interact with the amygdala, but for different reasons: ACC–amygdala for social valuations and OFC–amygdala for nonsocial, foraging valuations [96]. They also show that separable amygdala–PFC pathways perform social versus nonsocial functions (Fig. 5). Still, there is hardly a complete separation of ACC and OFC contributions to social and nonsocial processing, respectively. For example, OFC contributes to social behavior (e.g., ref [195]) and ACC contributes to object valuation and choice (eg., ref [140, 229]); future studies will need to understand the nature of the social and nonsocial contributions made by each area.

Emerging work has used social group size as a summary indicator of social behaviors. In both humans and nonhuman primates, social group size is positively correlated with estimates of gray matter volume in the amygdala and in cortex in the banks of the superior temporal sulcus (STS) [230, 231]. There is some evidence that functional and structural connectivity between ACC and anterior temporal lobe (including amygdala) varies with social group size in humans [232]. In addition, there is increased functional connectivity between cortex in the STS and ACC in macaques viewing video clips of social interactions that are ambiguous (as opposed to affiliative or aggressive) [233]. These findings suggest a close interaction among the amygdala, cortex in the banks of the STS, and ACC in social communication like that described earlier among amygdala, ITC/PRC and OFC for foraging.

## Summary and conclusion

Amygdala–PFC interactions are relevant to a range of survival-relevant behaviors in primates, including humans. The amygdala contributes a valuation element to representations of biological importance, updated in accord with current needs. The specifics of this influence depend on what an amygdala-connected area represents: objects or actions associated with specific outcomes; threats posed by predators or irritants; or social signals sent by conspecifics. Damage to the amygdala removes or impairs these influences, so behavior tends to lack its special relationship with the most biologically significant stimuli and their value at any given moment.

In the natural habitat of many extant primates, and probably ancestral primates as well, optimal foraging under risky conditions requires sensitivity to the current value of available resources predicted by visual information, along with estimates of moment-to-moment predation threats. Because anthropoid social systems provide protection from predators, especially during foraging expeditions, learning from the experience of conspecifics and making choices that enhance the foraging success of conspecifics both provide benefits to individuals in terms of inclusive fitness. Evidence shows that amygdala–PFC interactions contribute to all these behaviors.

More specifically, the PFC represents recently acquired information regarding the current desirability of nutrient outcomes (OFC) and the current availability of outcomes (VLPFC) that are linked to visual objects and other visual features, as well as to the current status of conspecifics (ACC) with respect to their dominance and social valuations. By representing features of the environment (e.g., of objects, contexts, or conspecifics) and their relationship to behavior, together with their up-to-date valuations, PFC regions—via their projections to the amygdala—make essential contributions to many aspects of motivated behavior. Specifically, PFC–amygdala projections promote emotions and motivations appropriate to the current situation. Applying this idea to the clinic requires surmounting the limitations of simple ideas limited to top-down control of “fear” responses. The more sophisticated concepts that promise to promote future progress will incorporate the many ways in which positively and negatively valenced outcomes influence the myriad emotions and motivations that characterize the affective life of primates.

## Future research directions

An extensive literature on the neural basis of internalizing psychopathologies suggests that aberrant amygdala–PFC interactions play a role in depression, anxiety, post-traumatic stress disorder, and phobias. Increasingly, neuroscience is providing tools to alter circuit function, whether through cognitive behavioral therapy or direct manipulation of neuronal activity. The findings reviewed here provide a foundation for a rational, evidence-based application of such tools. However, more work is needed to fully understand amygdala–PFC interactions, including the functional specializations among each PFC subregion in primates, the functional distinctions between amygdala-to-cortex influences and cortex-to-amygdala influences, and the relationship of these functions to the specific symptoms targeted by such treatments.
